# Silicon Improves Maize Photosynthesis in Saline-Alkaline Soils

**DOI:** 10.1155/2015/245072

**Published:** 2015-01-05

**Authors:** Zhiming Xie, Ri Song, Hongbo Shao, Fengbin Song, Hongwen Xu, Yan Lu

**Affiliations:** ^1^College of Life Sciences, Baicheng Normal University, Baicheng 137000, China; ^2^Northeast Institute of Geography and Agroecology, Chinese Academy of Sciences, Changchun 130102, China; ^3^College of Agronomy, Jilin Agricultural University, Changchun 130118, China; ^4^Key Laboratory of Coastal Biology & Bioresources Utilization, Yantai Institute of Coastal Zone Research, Chinese Academy of Sciences (CAS), Yantai 264003, China; ^5^School of Urban and Environmental Science, Huaiyin Normal University, Huai'an 223300, China

## Abstract

The research aimed to determine the effects of Si application on photosynthetic characteristics of maize on saline-alkaline soil, including photosynthetic rate (*P*_*n*_), stomatal conductance (*g*_*s*_), transpiration rate (*E*), and intercellular CO_2_ concentration (*C*_*i*_) of maize in the field with five levels (0, 45, 90, 150, and 225 kg·ha^−1^) of Si supplying. Experimental results showed that the values of *P*_*n*_, *g*_*s*_, and *C*_*i*_ of maize were significantly enhanced while the values of *E* of maize were dramatically decreased by certain doses of silicon fertilizers, which meant that Si application with proper doses significantly increased photosynthetic efficiency of maize in different growth stages under stressing environment of saline-alkaline soil. The optimal dose of Si application in this experiment was 150 kg·ha^−1^ Si. It indicated that increase in maize photosynthesis under saline-alkaline stress took place by Si application with proper doses, which is helpful to improve growth and yield of maize.

## 1. Introduction

Salinity and sodicity toxicities are worldwide agricultural and ecoenvironmental problems; it is estimated that there is approximately 27 million hectares of salinised soils in China's coastal and inland areas [1]. The saline and sodic soils, among which about 23% of the cultivated lands are saline and 37% are sodic, cover about 10% of total arable lands worldwide [2]. This kind of soil is widespread in arid and semiarid regions of the world and causes severe environmental and agricultural problems [3]. Soil salinity and sodicity, which seriously affect the stages of germination, seedling growth and vigour, vegetative growth, flowering, and fruit set of crops [4], adversely affect crop production in different regions, especially in arid and semiarid regions of the world [5, 6].

Supplement silicon, which is much cheaper than other methods to minimize salinity and sodicity, such as reclamation, water, and drainage, is an alternative way for overcoming the negative effects of salinity and sodicity on the plant growth and yield [7]. The beneficial effect of silicon is more evident under stress conditions; this is because silicon is able to protect plants from multiple abiotic and biotic stresses [8]. Silicon can alleviate the adverse effects of salt stress on plants by increasing cell membrane integrity and stability through its ability to stimulate the plants' antioxidant system [9]. Silicon application can moderate the salinity and sodicity stress in plants and plays a multitude of roles in plant existence and crop performance, and silicon is deposited in leaves leading towards decreased transpiration and hence dilutes salts accumulated in saline environment [10]. In general, graminaceous plants accumulate much more silicon in their tissues than other species [11].

Maize (*Zea mays *L.) is reported as salt susceptible [12]. This study aimed to investigate the effects of silicon fertilizer, which was conducted in field tests, on photosynthetic characteristics of maize under the condition of saline-alkaline soil in Northeast China.

## 2. Materials and Methods

### 2.1. Experimental Site and Soil Condition

During May to October, 2012, the field experiments were carried out in Agricultural Research Center in Gaoping Village, Baicheng City, Jilin Province, China (45.38°N latitude, 122.50°E longitude), on an saline-alkaline soil, where the climatic conditions are as follows: continental monsoon climate, lying in midtemperate zone, a mean annual temperature being 4.9°C, the annual sunshine time being 2, 919.4 hours, an average frost-free period being 157 days, and the annual average precipitation being 399.9 mm with 88% distributed in May–September. The basic properties of the soil (0–20 cm), which is saline-alkaline soil, are that it contains organic matter 19.6 g·kg^−1^, available nitrogen 112.46, available phosphorus 11.38 mg·kg^−1^, available potassium 162.39 mg·kg^−1^, available silicon 238.60 mg·kg^−1^, ESP 11.36%, EC 9.88 dS·m^−1^, K^+^ 83.62 mg·kg^−1^, Na^+^ 405.76 mg·kg^−1^, Ca^2+^ 43.26 mg·kg^−1^, Mg^2+^ 65.68 mg·kg^−1^, Cl^−^ 69.18 mg·kg^−1^, HCO_3_^−^ 896 mg·kg^−1^, SO_4_^2−^ 40.75, and pH 8.00.

### 2.2. Experimental Design

The experiment was conducted in the form of randomized complete block design (RCBD) with three replications in a 5 × 10 m^2^ net plot size. Maize (Zhengdan 958) was sown on May 6 with a density of 65 000 plants·ha^−1^. A uniform dose of basal fertilizer was applied to all experimental plots prior to seed sowing with N 200 kg·ha^−1^ as urea, P_2_O_5_ 100 kg·ha^−1^ as single super phosphate, and K_2_O 80 kg·ha^−1^ as potassium sulphate. Treatments were five levels of SiO_2_: T1 with SiO_2_ 0 kg·ha^−1^ (control), T2 with SiO_2_ 45 kg·ha^−1^, T3 with SiO_2_ 90 kg·ha^−1^, T4 with SiO_2_ 150 kg·ha^−1^, and T5 with SiO_2_ 225 kg·ha^−1^. The silicon fertilizer used, which was in the form of a sodium metasilicate (Na_2_SiO_3_·H_2_O) with the content of soluble SiO_2_ 30%, was produced by Yubei Fertilizer Company Limited, Xinxiang City, Henan Province, China. All fertilizers were applied as basal applications. In this experiment, maize was evaluated for its physiological parameters of net photosynthetic rate (*P*_*n*_), transpiration rate (*E*), stomatal conductance (*g*_*s*_), and intercellular CO_2_ concentration (*C*_*i*_). Observations were recorded at four key growth stages: big trumpet stage (or the 12-leaf stage), silking stage, grain filling stage, and milk stage. The dates of growth stages are shown in Table 1.

### 2.3. Measurement of Gas Exchange Parameters

The photosynthetic characteristics of gas exchange parameters, *P*_*n*_, *E*, *g*_*s*_, and *C*_*i*_ of the top second fully expanded leaf at the four growth stages in the field, were measured with a portable open flow gas exchange system LI-6400 (LI-COR Inc., USA) between 9:00 am and 11:00 am. The photosynthetically active radiation was 2000 *μ*mol·m^−2^·s^−1^, CO_2_ concentration was 350 *μ*mol·mol^−1^, and leaf temperature was 25°C [13–15].

### 2.4. Statistical Analysis

All the data were analyzed with one-way analysis of variance (ANOVA) procedures using SPSS Version 17.0 for Windows. The differences between means were compared by Duncan's test at 0.05 significance level.

## 3. Results

Observed from Tables 2, 3, and 4, changes in parameters of net photosynthetic rate (*P*_*n*_), transpiration rate (*E*), and stomatal conductance (*g*_*s*_) show that the values measured of these parameters from big trumpet stage to milk stage increased first from the beginning stage, big trumpet stage, and reached the peak values at silking stage, after which these values decreased as maize growing. According to the results of Table 5, there is a similar changing pattern among these Si application treatments T1, T2, T3, T4, and T5, where the values of intercellular CO_2_ concentration (*C*_*i*_) under the five Si treatments continued decreasing from big trumpet stage, got the lowest values of *C*_*i*_ at grain filling stage, and then slowly increased at milk stage.

### 3.1. Net Photosynthetic Rate (*P*_*n*_)

Results showed that the parameter photosynthetic rate (*P*_*n*_) in leaves of maize was affected significantly by Si application treatments and *P*_*n*_ was significantly decreased in later growth stages (Table 2). From big trumpet stage to milk stage, the values of *P*_*n*_ under the treatments of T3, T4, and T5 (90 kg·ha^−1^ Si, 150 kg·ha^−1^ Si, and 225 kg·ha^−1^ Si) in the same growth stage were significantly (*P* ≤ 0.05) higher than those under the treatment of T1 and T2 (0 kg·ha^−1^ and 45 kg·ha^−1^ Si). When the content of Si application reached 90 kg·ha^−1^ Si, the value of *P*_*n*_ increased significantly (*P* ≤ 0.05) with the increased dose of Si application. Low level of Si application (T2, 45 kg·ha^−1^ Si) did not change *P*_*n*_ significantly and high levels of Si application (T4, 150 kg·ha^−1^ Si, and T5, 225 kg·ha^−1^ Si) increased *P*_*n*_ significantly (*P* ≤ 0.05), but there were no significant differences between the treatments of 150 kg·ha^−1^ Si and 225 kg·ha^−1^ Si dose.

### 3.2. Transpiration Rate (*E*)

During each stage from big trumpet stage to milk stage, transpiration rate (*E*) (Table 3) got higher values under the treatments of T1 (without Si application) and T2 (45 kg·ha^−1^ Si) than those under the treatments of T3, T4, and T5. There were no significant differences between T1 and T2. The values of *E* significantly (*P* ≤ 0.05) decreased with the increase of Si applying dose from 90 kg·ha^−1^ Si in each growth stage. Comparing the values of *E* by Si application of T3, T4, and T5 with those by T1, it is shown that, during big trumpet stage, the former decreased 16.28%, 20.32%, and 20.79%, respectively, compared to those of the latter; during silking stage, the former decreased 9.44%, 15.63%, and 16.75%, respectively, compared to those of the latter; during grain filling stage, the former decreased 11.38%, 21.12%, and 22.89%, respectively, compared to those of the latter; during milk stage, the former decreased 18.33%, 29.70%, and 31.32%, compared to those of the latter. There were no significant differences between the treatments of T1 and T2. So during the four studied stages, the value of *E* of maize began to decreased significantly (*P* < 0.05) when the dose of Si application got to the amount of 90 kg·ha^−1^, after which the values of *E* decreased dramatically with the increased dose of Si application; there was no significant difference between the treatments of doses 150 kg·ha^−1^ and 225 kg·ha^−1^.

### 3.3. Stomatal Conductance (*g*_*s*_)

According to the data (Table 4) of big trumpet stage and silking stage, it is shown that the values of stomatal conductance (*g*_*s*_) of maize were increased significantly (*P* ≤ 0.05) at higher Si levels of T4 and T5 (150 kg·ha^−1^ and 225 kg·ha^−1^) as compared to lower levels of Si application of T1, T2, and T3 (0 kg·ha^−1^, 45 kg·ha^−1^, and 90 kg·ha^−1^). There were no significant differences between the treatments of T4 and T5 as well as among the treatments of T1, T2, and T3. The data (Table 4) showed that, at grain filling stage and milking stage, the values of *g*_*s*_ of maize significantly (*P* < 0.05) increased by Si application at levels of 90 kg·ha^−1^ (T3), 150 kg·ha^−1^ (T4), and 225 kg·ha^−1^ (T5); the values of *g*_*s*_ of maize were significantly enhanced with the increased dose of Si application of 90 kg·ha^−1^; there were significant differences between T3 and T4, and there was no significant difference between T4 and T5. So at big trumpet stage and silking stage, the value of *g*_*s*_ of maize began to increase significantly (*P* < 0.05) when the dose of Si application got to the amount of 150 kg·ha^−1^; there was no significant difference in the values of *g*_*s*_ of maize between the treatments of doses 150 kg·ha^−1^ and 225 kg·ha^−1^; at grain filling stage and milk stage, the value of *g*_*s*_ of maize began to increase significantly (*P* < 0.05) when the dose of Si application got to the amount of 90 kg·ha^−1^, after which the values of *g*_*s*_ of maize were enhanced significantly with the increased dose of Si application; there was no significant difference between the treatments of doses 150 kg·ha^−1^ and 225 kg·ha^−1^.

### 3.4. Intercellular CO_2_ Concentration (*C*_*i*_)

From big trumpet stage to milk stage, the data (Table 5) showed that the values of intercellular CO_2_ concentration (*C*_*i*_) of maize with the Si treatments of T3, T4, and T5 were significantly (*P* ≤ 0.05) higher than those with T1 and T2; there was no significant difference between the treatments of T1 and T2. The values of *C*_*i*_ began to increase significantly (*P* ≤ 0.05) when the dose of Si application got to the amount of 90 kg·ha^−1^, after which the values of *C*_*i*_ were dramatically enhanced with the increased dose of Si application; there were significant (*P* ≤ 0.05) differences among the treatments of T3, T4, and T5. During big trumpet stage, comparing the values of *C*_*i*_ by Si application of T3, T4, and T5 with those by T1, it is shown that the former increased 13.1%, 17.1%, and 26.3%, respectively, compared to those of the latter; during silking stage, the former increased 16.6%, 20.6%, and 30.6%, respectively, compared to those of the latter; during grain filling stage, the former increased 14.1%, 26.5%, and 35.6%, respectively, compared to those of the latter; during milk stage, the former increased 13.5%, 25.5%, and 33.6% compared to those of the latter. There was no significant difference between the treatments of T1 and T2. So in these four studied stages, the value of *C*_*i*_ of maize began to increase significantly (*P* < 0.05) when the dose of Si application got to the amount of 90 kg·ha^−1^, after which the values of *C*_*i*_ increased dramatically with the increase of dose of Si applying.

## 4. Discussion

Under abiotic stresses such as toxicity, salinity, and lodging, silicon is reported to improve the growth of many kinds of higher plants [16]. Silicon can improve the growth of plants under salinity stress [17]. Exogenously applied Si significantly increased photosynthetic efficiency (*A*), stomatal conductance (*g*_*s*_) and increased internal CO_2_ concentration (*C*_*i*_) in maize under saline conditions [18, 19]. Our results showed that, under the condition of saline-alkaline soil, the values of *P*_*n*_, *C*_*i*_, and *g*_*s*_ of maize leaves were significantly enhanced by Si application and that of *E* was decreased with the increase of Si supplied; similar improvement was reported in crops of strawberry, maize, Chinese cabbage, and rice [20–23]. The main mechanisms of improving crops growth by silicon lie in its functions of stimulation of photosynthesis, reduction of plant transpiration rate, and enhancement of tissue strength [24]. Our researches showed that, in the four studied growth stages, the values of *P*_*n*_ by Si application were significantly enhanced compared with those with no Si application. Similar results were reported that addition of Si can enhance the photochemical efficiency of plants under salt stress [25]. Photosynthetic capacities of crops treated by Si application can be improved because the size of chloroplasts is enlarged and the number of grana in leaves is increased [26]. Researches on crops of barely (*Hordeum vulgare* L.), rice (*Oryza sativa* L.), sugarcane (*Saccharum officinarum* L.), and wheat (*Triticum aestivum* L.) showed that silicon deposited in leaves is helpful to improve the potential and efficiency of photosynthesis by opening angle of leaves, decreasing self-shading, and keeping the leaf erect [27], which play important roles in increasing *P*_*n*_ of crops.

According to our research, during the studied growth stages, the value of *E* of maize began to decrease significantly (*P* < 0.05) at the dose of 90 kg·ha^−1^ Si application, and above this dose of Si application the values of *E* decreased dramatically with the increased dose of Si application. By Si application, plant's internal water stress can be reduced; therefore, salt stress can be withstood as the rate of transpiration being influenced by the amount of Si gel associated with the cellulose in the cell walls of epidermal cells [28]. Similar results showed that water loss in maize can by reduced by Si application because Si can change the morphological structures of leaf epidermal cell [29]. Si is deposited in leaves leading towards decreased transpiration and hence dilutes salts accumulated in saline environment [30]. Unnecessary water loss can be limited through the epidermis, which is double layer, and silica combines with cellulose in the epidermal cells of leaf blade [31]. Another reason to explain the decrease of *E* is that the stomata opening can be influenced by Si [32]. Salt-stressed plants supplied with Si showed values of WUE 17% greater than those of salinized plants which were not supplied with Si by reducing the transpiration [30].

In our research, the value of *g*_*s*_ of maize began to increase significantly when the dose of Si application got to the amount of 150 kg·ha^−1^ at big trumpet stage and silking stage; the value of *g*_*s*_ of maize began to increase significantly when the dose of Si application got to the amount of 90 kg·ha^−1^, after which the values of *g*_*s*_ of maize were enhanced significantly with the increased dose of Si application at grain filling stage and milk stage. Similar reports on rice (*Oryza sativa* L.) are that Si application can enhance the stomatal conductance of rice plants subjected to salt stress, which shows silicate can reduce Na uptake via decline in the transpiration rate [30]. Silicon added to the saline growth medium improved photosynthetic activity [18, 19]. Si amendment enhanced the stomatal conductance of rice plants subjected to salt stress showing that silicate can reduce Na uptake via decline in the transpiration rate, which ultimately results into the reduction in growth and net photosynthesis [10]. It was also reported that *g*_*s*_ can be increased by Si fertilizer, because the increased *g*_*s*_ can regulate gas exchange, increase CO_2_ uptake, and subsequently improve the capacity and efficiency of photosynthesis [18, 19, 33–39].

In the four studied growth stages, the value of *C*_*i*_ of maize began to increase significantly at the dose of 90 kg·ha^−1^ Si application, after which the values of *C*_*i*_ increased dramatically with the increase of dose of Si application, which showed that photosynthetic efficiency of leaves can be enhanced by Si application [18]. Si applying can prevent the activities of photosynthetic enzymes in mesophyll cells from decreasing [34]. Si application reduces the transpiration rate to restrict the Na uptake; as a result, CO_2_ intake is enhanced [10]. Under salt stress condition, the double membranes of chloroplasts disappeared, but membrane integrity was markedly improved in the salt treatment supplemented with Si [35]. The values of *C*_*i*_ can be increased dramatically by Si application under salt stress, which is stressful environment with the accumulation of ROS, such as superoxide radicals (O_2_^∙−^), hydroxyl radicals (OH^−^), and hydrogen peroxide (H_2_O_2_), and the activity of defense system affected by salinity stress may be enhanced by Si application [10, 19, 36–43].

## 5. Conclusion

Silicon plays an important role to enhance photosynthesis ability and efficiency of plants under salinity stress [10]. The field research showed that values of photosynthetic rate (*P*_*n*_), stomatal conductance (*g*_*s*_), transpiration rate (*E*), and intercellular CO_2_ concentration (*C*_*i*_) of maize plants in saline-alkaline soil were affected by Si application and salinity stress can be alleviated by Si application. In our research, the optimal dose of Si application on saline-alkaline soil was 150 kg·ha^−1^, under which photosynthetic ability of maize was greatly increased at studied growth stages. So in this research, increase in maize photosynthesis under saline-alkaline stress took place by Si application with proper doses, with which it is helpful to improve growth and yield of cereal crops [29].

## Figures and Tables

**Table 1:** Dates of growth stages of maize on saline-alkaline soil in 2012.

Sowing stage	Emergence stage	Big trumpet stage	Silking stage	Grain filling stage	Milk stage
4/26	5/20	7/12	8/2	8/16	9/16

**Table 2:** Effects of Si application on net photosynthetic rate (*P*_*n*_) in leaves of maize.

Growth stages	Photosynthetic rate (*P*_*n*_) (*μ*mo1·m^−2^·s^−1^)				
	T1	T2	T3	T4	T5
Big trumpet stage	19.15 ± 0.25^c^	18.86 ± 0.69^c^	23.02 ± 0.73^b^	24.83 ± 0.75^a^	24.95 ± 0.42^a^
Silking stage	33.62 ± 0.86^c^	34.11 ± 0.62^c^	37.86 ± 0.75^b^	39.30 ± 0.55^a^	39.52 ± 0.39^a^
Grain filling stage	24.11 ± 0.33^c^	23.93 ± 0.50^c^	26.10 ± 0.28^b^	28.35 ± 0.37^a^	28.67 ± 0.47^a^
Milk stage	12.70 ± 0.59^c^	12.91 ± 0.36^c^	14.32 ± 0.53^b^	16.85 ± 0.40^a^	16.89 ± 0.33^a^

Means (±SD) labeled with different letters within each column are significantly different (*P* < 0.05) by Duncan's test, *n* = 10.

**Table 3:** Effects of Si application on transpiration rate (*E*) in leaves of maize.

Growth stages	Transpiration rate (*E*) (mmol·m^−2^·s^−1^)				
	T1	T2	T3	T4	T5
Big trumpet stage	8.66 ± 0.21^a^	8.23 ± 0.16^a^	7.25 ± 0.10^b^	6.90 ± 0.12^c^	6.86 ± 0.09^c^
Silking stage	9.85 ± 0.08^a^	9.67 ± 0.18^a^	8.92 ± 0.15^b^	8.31 ± 0.13^c^	8.20 ± 0.16^c^
Grain filling stage	7.82 ± 0.15^a^	7.97 ± 0.11^a^	6.93 ± 0.13^b^	6.09 ± 0.12^c^	6.03 ± 0.09^c^
Milk stage	4.31 ± 0.08^a^	4.15 ± 0.10^a^	3.52 ± 0.07^b^	3.03 ± 0.08^c^	2.96 ± 0.05^c^

Means (±SD) labeled with different letters within each column are significantly different (*P* < 0.05) by Duncan's test, *n* = 10.

**Table 4:** Effects of silicon on stomatal conductance (*g*_*s*_) in leaves of maize.

Growth stages	Stomatal conductance (*g*_*s*_) (mol·m^−2^·s^−1^)				
	T1	T2	T3	T4	T5
Big trumpet stage	0.39 ± 0.06^b^	0.37 ± 0.08^b^	0.40 ± 0.02^b^	0.57 ± 0.09^a^	0.56 ± 0.07^a^
Silking stage	0.66 ± 0.03^b^	0.63 ± 0.05^b^	0.65 ± 0.06^b^	0.75 ± 0.08^a^	0.78 ± 0.09^a^
Grain filling stage	0.47 ± 0.08^c^	0.45 ± 0.03^c^	0.58 ± 0.07^b^	0.66 ± 0.05^a^	0.69 ± 0.06^a^
Milk stage	0.23 ± 0.02^c^	0.25 ± 0.03^c^	0.32 ± 0.02^b^	0.39 ± 0.03^a^	0.38 ± 0.01^a^

Means (±SD) labeled with different letters within each column are significantly different (*P* < 0.05) by Duncan's test, *n* = 10.

**Table 5:** Effects of silicon on intercellular CO_2_ concentration (*C*_*i*_) in leaves of maize.

Growth stages	Intercellular CO_2_ concentration (*C*_*i*_) (μmo1·mol^−1^)				
	T1	T2	T3	T4	T5
Big trumpet stage	139.10 ± 1.73^d^	138.23 ± 2.02^d^	157.32 ± 1.35^c^	162.95 ± 2.13^b^	175.68 ± 2.23^a^
Silking stage	116.82 ± 1.69^d^	120.36 ± 1.86^d^	136.20 ± 1.57^c^	140.85 ± 1.86^b^	152.61 ± 1.75^a^
Grain filling stage	103.73 ± 1.93^d^	108.92 ± 1.70^d^	118.36 ± 2.33^c^	131.25 ± 1.78^b^	140.62 ± 1.66^a^
Milk stage	106.90 ± 1.28^d^	112.78 ± 1.66^d^	121.32 ± 1.51^c^	134.25 ± 1.36^b^	142.83 ± 1.92^a^

Means (±SD) labeled with different letters within each column are significantly different (*P* < 0.05) by Duncan's test, *n* = 10.
